# Supplementary material for: Scd1 Deficiency in Early Embryos Affects Blastocyst ICM Formation through RPs-Mdm2-p53 Pathway
Source: Int J Mol Sci. 2023 Jan 16;24(2):1750. doi: 10.3390/ijms24021750 (PMC9864350; doi:10.3390/ijms24021750)
Supplement: Supplementary file 1 [file ijms-24-01750-s001.zip › ijms-2136253-supplementary.pdf]

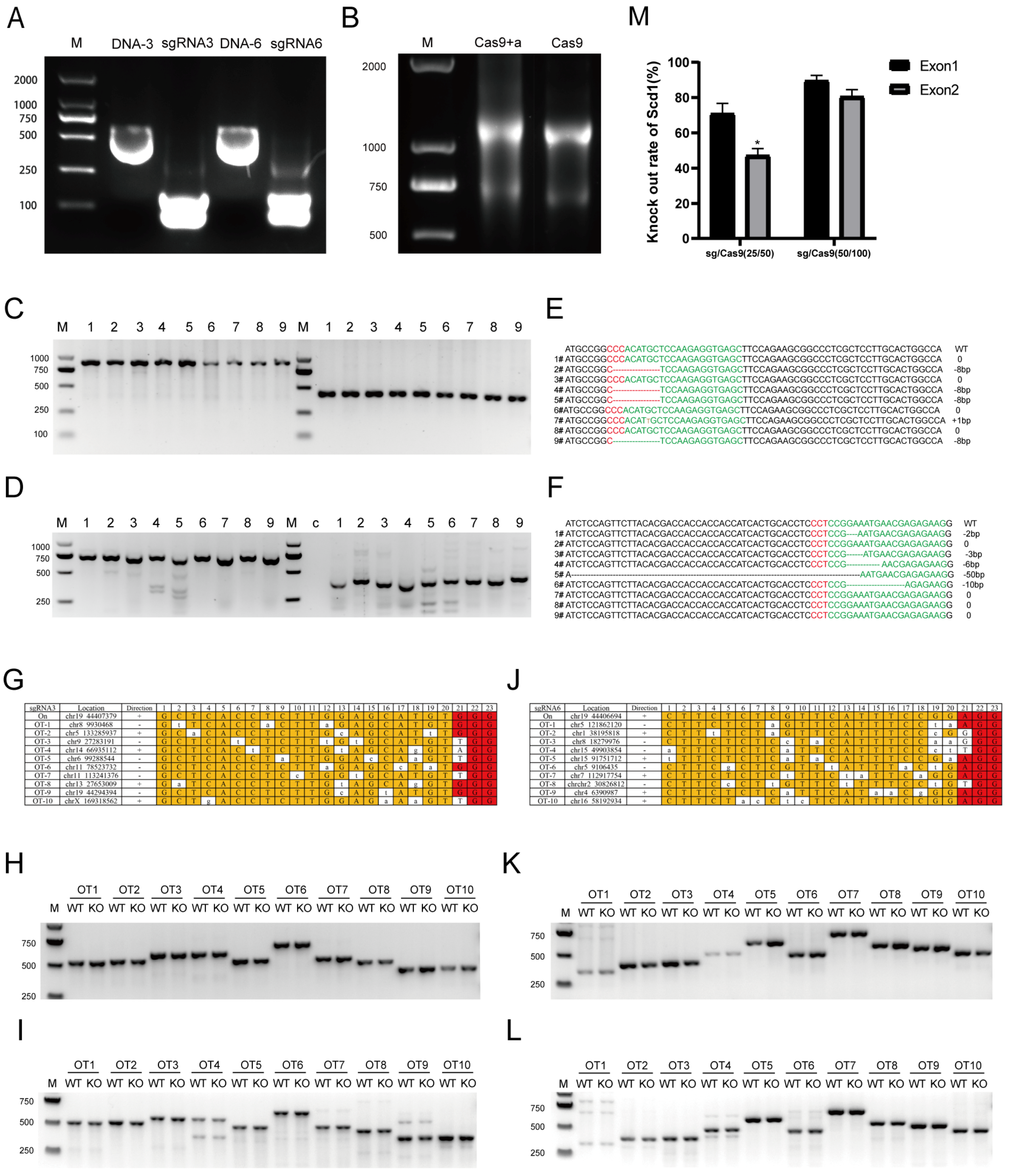

Figure S1. Preparation of high efficiency sgRNA/Cas9 mRNA. (A): Preparation of sgRNA3 and sgRNA6. M: Marker; DNA-3 and DNA-6 were the templets of invitro transcription for sgRNA3 and sgRNA6 generation respectively. sgRNA3 and sgRNA6 were finally sgRNAs for microinjection. (B): Preparation of Cas9 mRNA. M: Marker; Cas9 was the mRNA after in vitro transcription, and Cas9+a was the finally Cas9mRNA adding polyA tail for microinjection. (C): PCR amplification of Exon1 (404bp) and Exon2 (719bp) sites for each mouse embryos. (D): T7 Endonuclease I (T7EN1) digestion result of embryos for both two exons after microinjection. (E): Sequencing detection of exon1 and (F): exon2 regions. 1#-9# were numbers of detected embryos. (G-L): High-probability targeting site information of sgRNA3 (G) and sgRNA6 in mouse genome (J). Predicted off-target site amplification of sgRNA3 (H) and sgRNA6 (K). T7EN1 digestion for sgRNA3(I) and sgRNA6 (L) off-target sites. ON: Target site; TO1-10: off target sites; WT: wild type; the yellow highlight represents a coincident target sequence, and the red highlight represents a coincident PAM sequence. knock out efficiency of two different sgRNA/Cas9mRNA concentration at Exon1and Exon2. (M): Comparation of *Scd1* gene knock out efficiency between exon1 and exon2 with two different concentrations of sgRNA/Cas9. The knockout rate equals to the proportion of knockout embryos in total detected embryos. Two-tailed Student's t-test was used for statistical analysis, * $P < 0.05$.

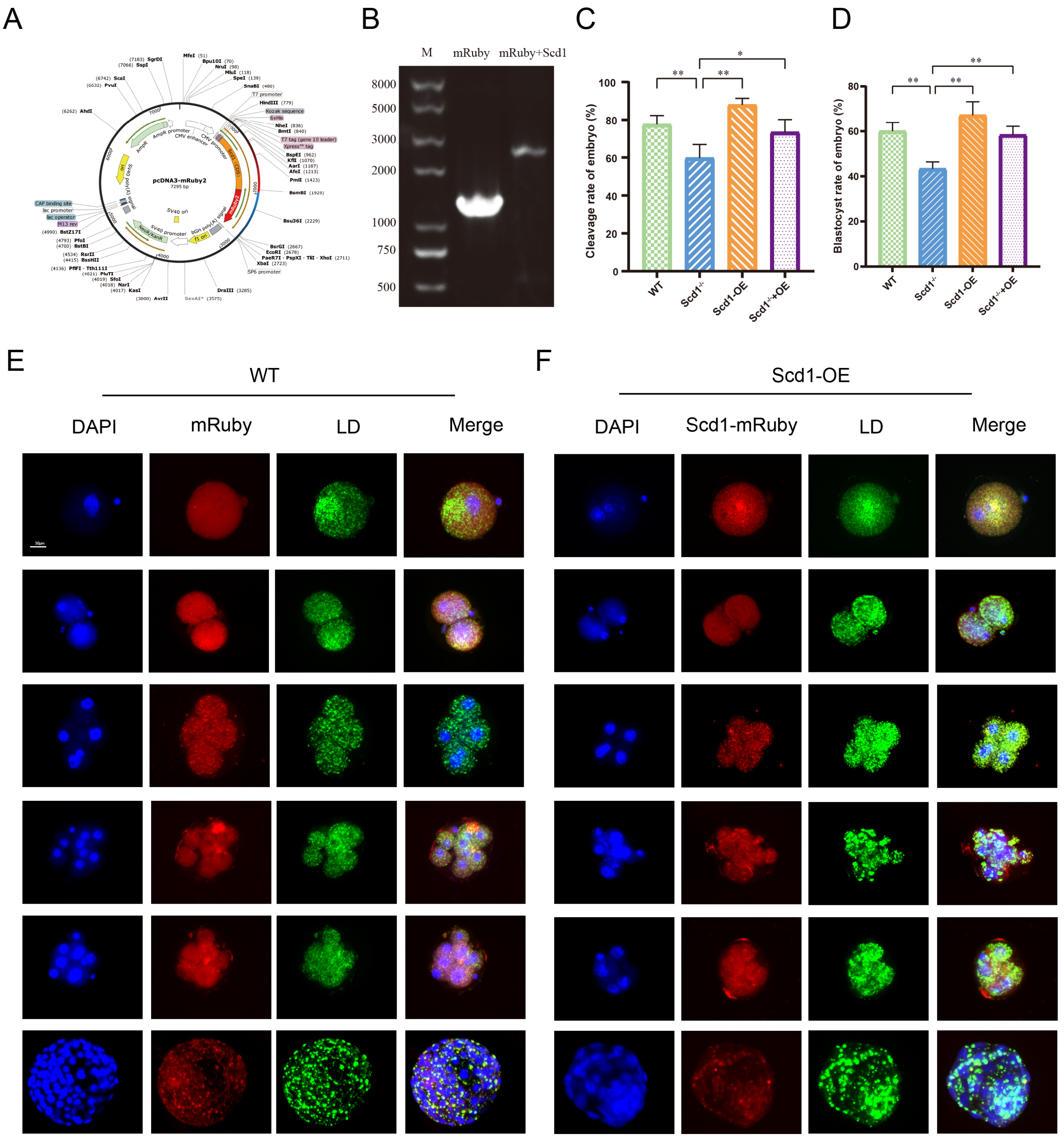

Figure S2. Gain of Scd1 reverses embryonic development and lipid droplet generation. (A): Plasmid mapping for *Scd1* overexpression. (B): The PCR fragment of mRuby and mRuby-Scd1 that were the templet of in vitro transcriptional. M: *Trans*2K® Plus II DNA Marker, mRuby+Scd1: DNA fragment containing mRuby: 1296bp, mRuby: DNA fragment containing mRuby-Scd1:2361bp. (C): The cleavage rate of embryos after *Scd1* overexpression in WT and *Scd1*$^{-/-}$ embryos. (D): The blastocyst rate of embryos after *Scd1* overexpression in WT and *Scd1*$^{-/-}$ embryos. ** $P < 0.01$, * $P < 0.05$. (E): Lipid droplet fluorescence image of WT embryos injected with mRuby mRNA. (F): Lipid droplet fluorescence image of WT embryos injected with mRuby-Scd1 mRNA. Red: mRuby, green: lipid droplet. Bar, 30μm.

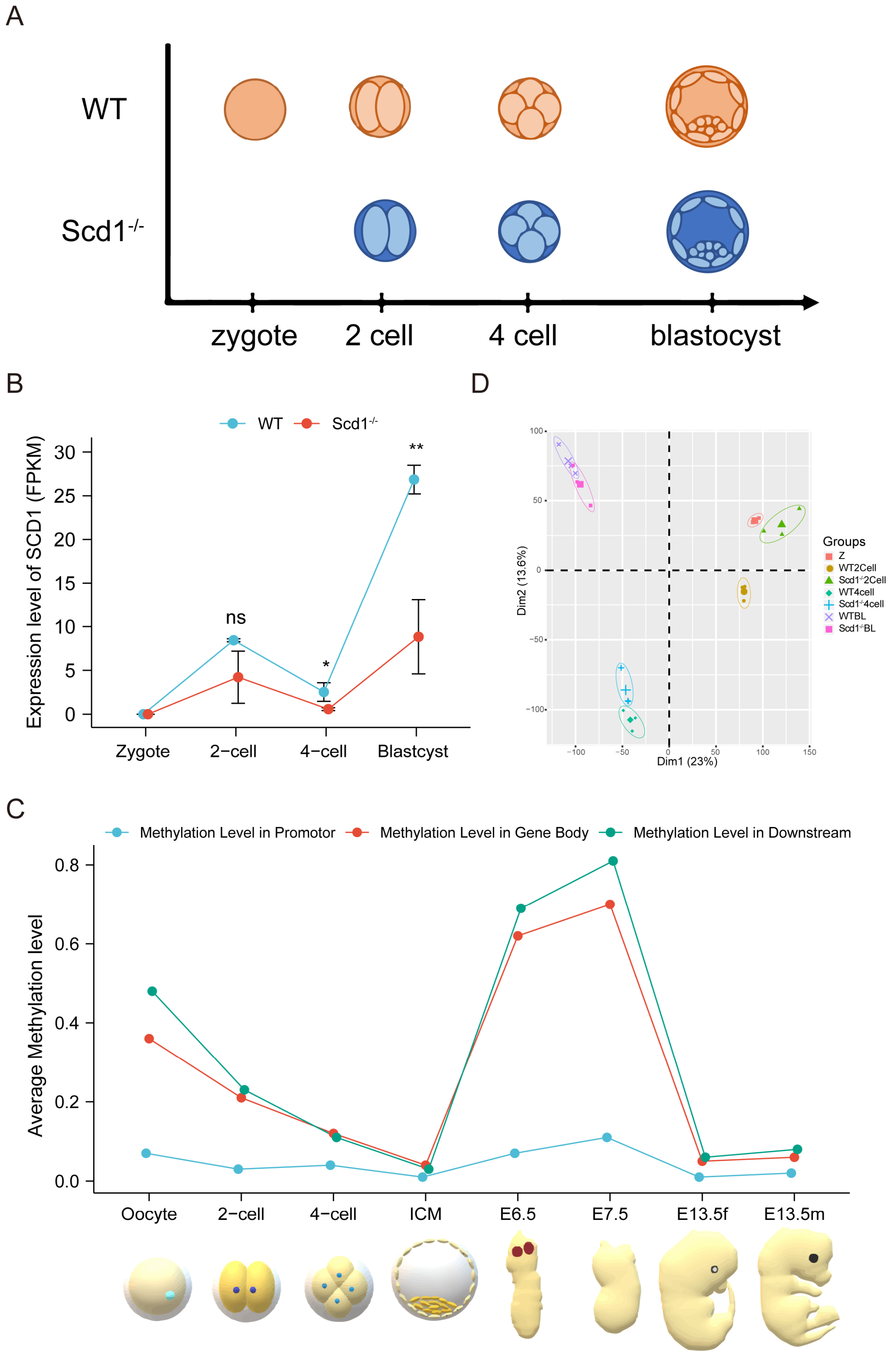

Figure S3. Single-embryo RNA-Seq atlas exhibits the increasing requirement of Scd1 during embryonic development. (A): Schematic diagram of embryonic single-cell sequencing samples, and three embryos were collected as repeats in each development stage in each group. (B): The expression level of *Scd1* gene during the embryo development. (C): The methylation level of *Scd*1 gene during embryo development. ** P < 0.01. (D): PCA analysis of all samples.

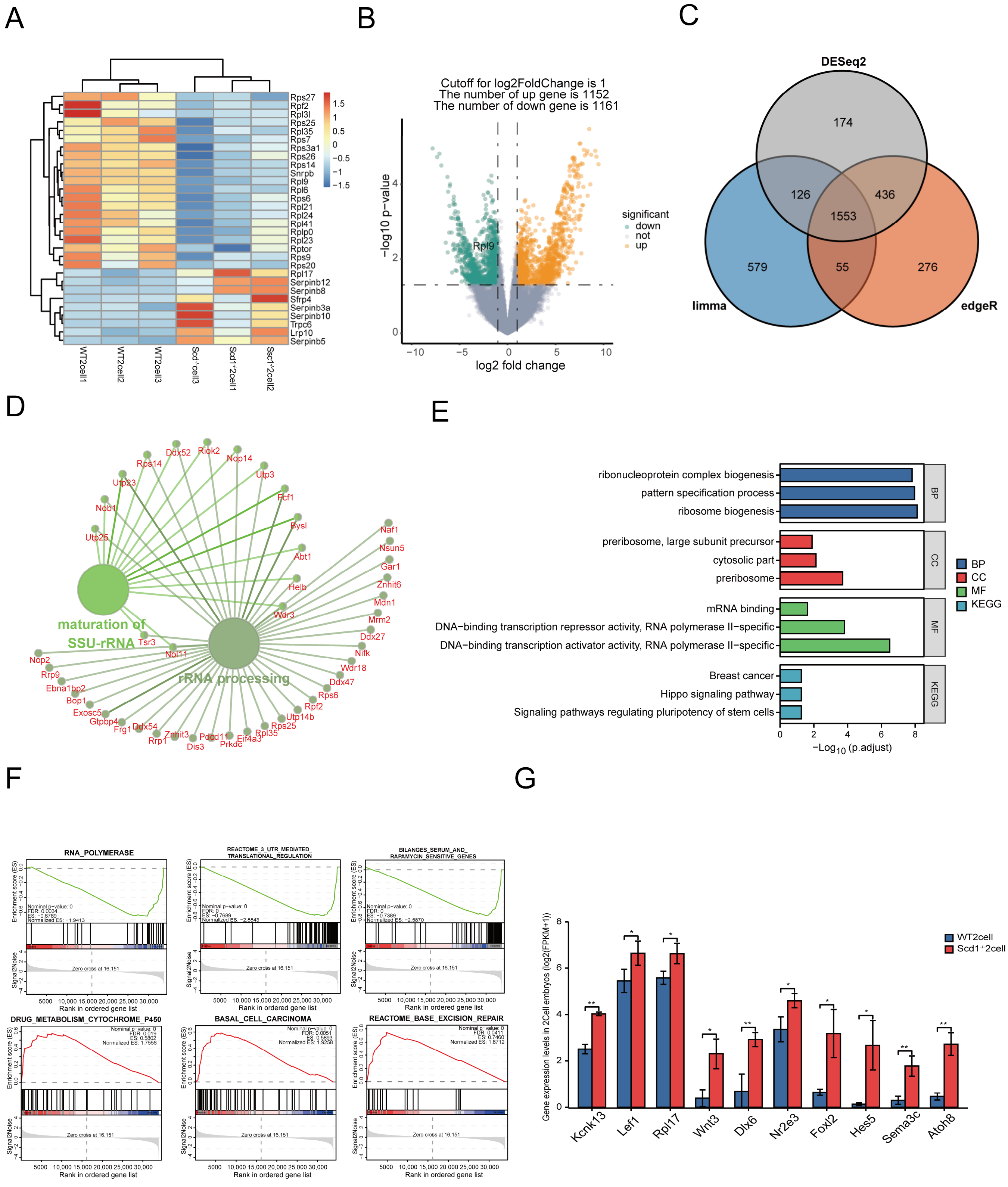

Figure S4. Ribosome biogenesis was suppressed in 2-cell stage $Scd^{1-/-}$ embryos. (A-C): Select the differential genes during 2-cell stage using three analysis methods. (A): heatmap of top 30 different DEGs. (B): Volcano map. (C): analysis of differential genes through three methods. (D): Functional enrichment analysis of differential genes. (E): GO and KEGG analysis of DEGs. (F): The GSES analysis for 2 cell stage embryos. (G): The expression level of up-regulated DEGs. * P < 0.05; ** P < 0.01.

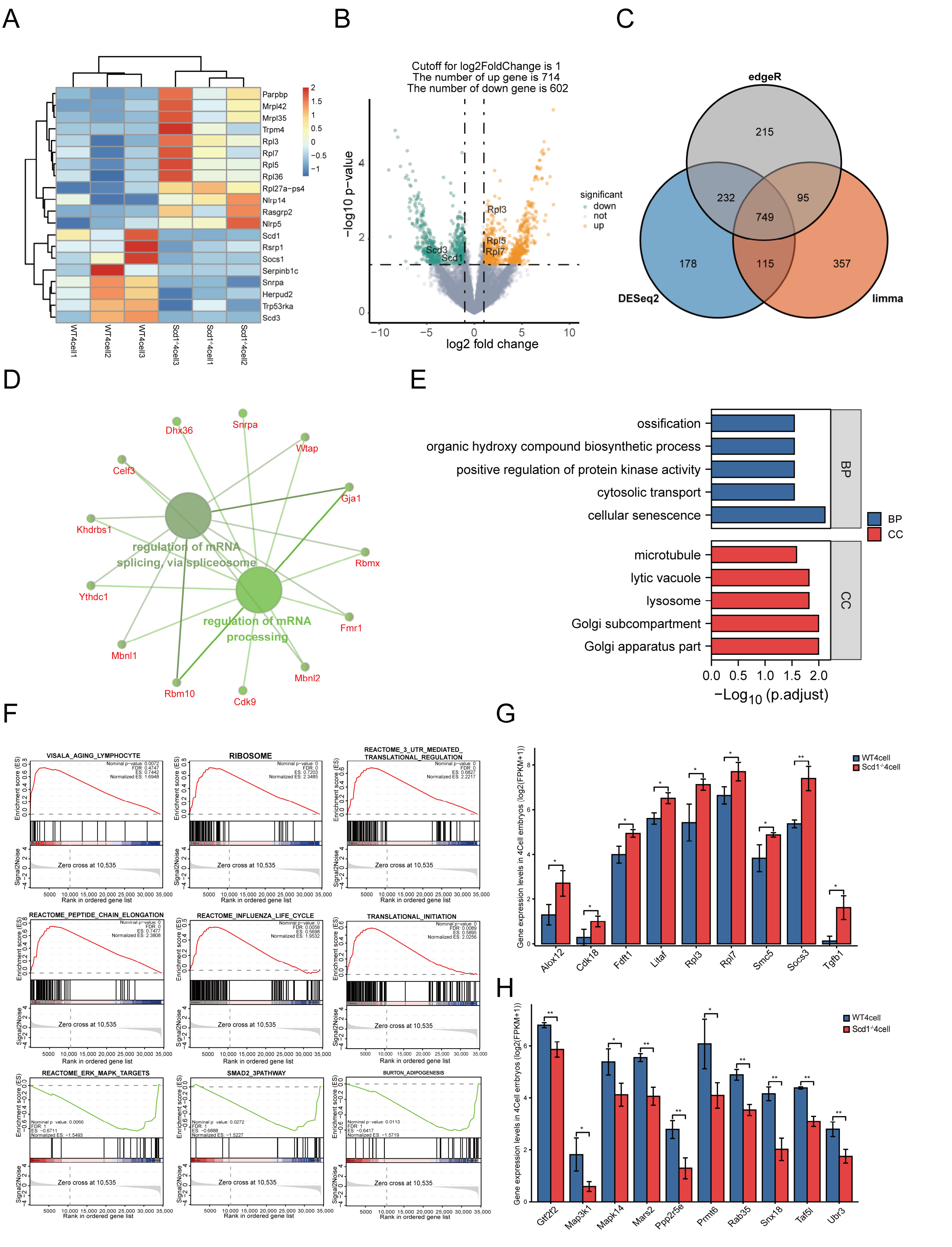

Figure S5. Ribosome biogenesis and RNA translation was reversely upregulated in 4-cell stage Scd1$^{-/-}$ embryos. (A-C): Selection of DEGs during 4-cell stage using three analysis methods. (A): heatmap of top 20 different DEGs. (B): Volcano map. (C): Venn analysis of DEGs through three methods. (D): Functional enrichment analysis of DEGs based on cytoscape. (E): GO and KEGG analysis of DEGs. (F): The GSEA analysis for sequencing result. (G): up-expression and H: down-expression of DEGs. * $P < 0.05$; ** $P < 0.01$.

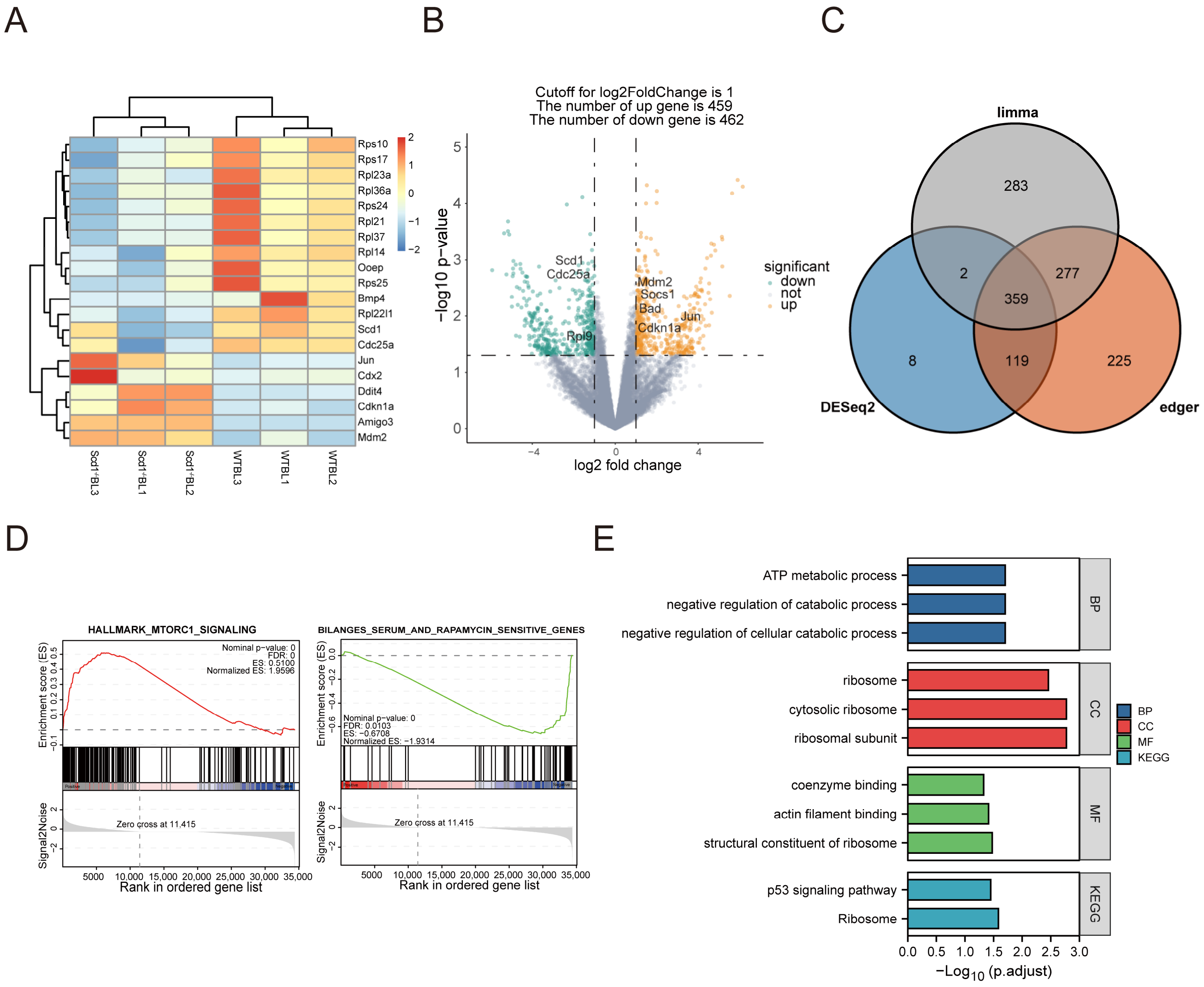

Figure S6. P53 pathway was stimulated in the Scd1$^{-/-}$ blastocyst. (A-C): Selection of DEGs during blastocyst stage using three analysis methods. (A): heatmap of top 20 different DEGs. (B): Volcano map. (C): Analysis of DEGs through three methods. (D): The GSEA analysis for sequencing result. (E): GO and KEGG analysis of DEGs at blastocyst stage.

Table S1. the sequence of primers used in the experiment.

Name	Forward sequence	Reverse sequence
Cdx2	CAAGGACGTGAGCATGTATCC	GTAACCACCGTAGTCCGGGTA
Gapdh	TGGATTTGGACGCATTGGTC	TTTGCACTGGTACGTGTTGAT
Sox2	GCGGAGTGGAAACTTTTGTCC	CGGGAAGCGTGTACTTATCCTT
Scd1	TCCCTCCGGAAATGAACGAG	TGCAGCAGGACCATGAGAAT
Bcl2	AAACCCTCCATCCTGTCC	TCCTAAACCCTGCTTCCC
Bax	CTGGATCCAAGACCAGGGTG	GTGAGGACTCCAGCCACAAA
Casp3	GAGCTTGGAACGGTACGCTA	GAGTCCACTGACTTGCTCCC
Pcna	TTCACAAAAGCCACTCCAC	TGCCTAAGATGCTTCCTCA
Rad18	TTTCTTAGCGGGAGCGT	GCTGCTTCTGAGGTTTGG
Brca1	CTAAAGAGCCAGCCCACA	TTATCAGCACCAGCAGCA
Trp53bp1	AACCCCGTGAAAACCCT	CACTTGAATGGTGCTGCTT
Trp53	CACAGCACATGACGGAGGTC	TCCTTCCACCCGGATAAGATG
Chek2	CCTGAGGACCAAGAACCTGA	CCGTCCTTCTCAACAGTGGT
Cdkn1a	AGATCCACAGCGATATCCAGAC	ACCGAAGAGACAACGGCACACT
IVT-sgRNA	TCTCGCGCGTTTCGGTGATGACGG	AAAAAAAGCACCGACTCGGTGCCACTTTTTC
IVT-mRuby	ACCAAAATCAACGGGACTTTC	TCTTCCCAATCCTCCCCCTTG
mE1	AATACTGAACACGGTCATCCCA	ACTCATCTGCCCAAATTACAATC
mE2	ATCTGTTTTCCGATGGTCTT	ACAGGGACTCAGTATTCATGTTA
3-OT1	TCTCGGGAGACAGATTGAGC	GAGGGGACAGAGGAAGTAGC
3-OT2	CCCAATGAAGCTGAAAACAC	CCACCAGTTGTGATCACTGA
3-OT3	AGGGCACTGCATGTGCATGT	TGAGCACCCTCATGTCTACC
3-OT4	GGCCTCAGTGATGGGTGAGA	AGGGAGGAGGAATACATTGA
3-OT5	TCGGCCACACATCAAGTAAG	CCTCGCAACATTCTGTAACT
3-OT6	TGGCCAAAAAAGTTGGATTG	AGGGTCACATCACTGACTCA
3-OT7	ACGGCCAAGGACTCCAGAAA	CCTCCCAACATGAACTTCTC
3-OT8	TCCCCATCCTTTCATGTGTT	TCCCCCACTCTTTACAAGAT
3-OT9	GCGGGCTGCAGAAACTTAGT	GCACCCTTAGCAACTCACAA
3-OT10	TTGGCTATAAAACAGTCACA	TCGGTGTAGCTGTTTGAAAT
6-OT1	AAACCCTGTCTCGAAAAACA	TTGCCATGATCAAATACTGA
6-OT2	TTCCCTGAATGCTACTGTCT	AAGGAGGCTGTACATGTGTG
6-OT3	ATGGCTCAAAGTCAATACCT	AGGCAACATATCCAAGTCTG
6-OT4	TCCCACCAGAATTTCTTAGA	GGTCACAGTTAAAATTATCT
6-OT5	TGGGATTCTGGAAGTGGTAA	GGGAACAGAGACAAGACACT
6-OT6	GTGGGATGCAATGAAAACTG	AGGCAGCAAAGACACAGAGA
6-OT7	AGGGAGGGATCACACTTCAG	CATCCCAGTTTGAACACTGT
6-OT8	TGGCCCTTCATCATTCCTGA	GCCTCTTGGTGCACAGTTCT
6-OT9	CCTGCCTCCAGTGAAGTTTT	TTGCCCACCAAGATAAGACA
6-OT10	CCCCACAGCCTCTTGATTCT	TGTCCTTCTCATTGTTGATC

Table S1 the sequence of primers used in the experiment
IVT-sgRNA represent the primers for PCR to product IVT templet from the pUC57 plasmid.
IVT-mRuby represent the primers in pcDNA3-mRuby2 plasmid for PCR to product Scd1-OE IVT templet
3-OT1-3-OT10 represent the primers for sgRNA3 off-target detection
6-OT1-3-OT10 represent the primers for sgRNA6 off-target detection
